# Smoking control in China: A need for comprehensive national legislation

**DOI:** 10.1371/journal.pmed.1004065

**Published:** 2022-08-25

**Authors:** Bo Xi, Costan G. Magnussen

**Affiliations:** 1 Department of Epidemiology, School of Public Health/Qilu Hospital, Cheeloo College of Medicine, Shandong University, Jinan, China; 2 Baker Heart and Diabetes Institute, Melbourne, Victoria, Australia; 3 Research Center of Applied and Preventive Cardiovascular Medicine, University of Turku, Turku, Finland; 4 Centre for Population Health Research, University of Turku and Turku University Hospital, Turku, Finland

## Abstract

In this Perspective, Bo Xi and Costan Magnussen discuss the trends in smoking prevalence in China reported by Mei Zhang and colleagues, and the need for effective tobacco control strategies.

Tobacco smoking is a leading cause of noncommunicable disease and premature death in China. An up-to-date understanding of smoking prevalence trends in China is important to guide government to establish tobacco control polices. In this issue of *PLOS Medicine*, Zhang and colleagues report the recent trends in tobacco smoking in mainland China from 2007 to 2018 [1]. Their data broaden our understanding of the diverse trends in tobacco smoking that are occurring in urban and rural regions, and across different geographical provinces/municipalities, within China.

Prior to this report by Zhang and colleagues, data from 2 national studies had also shown a decline in smoking prevalence in mainland China before 2013. Based on data from the China Health and Nutrition Survey (age range: ≥15 years), Li and colleagues reported that the prevalence of tobacco smoking had decreased among men from 60.6% in 1991 to 51.6% in 2011 and among women from 4.0% in 1991 to 2.9% in 2011 [2]. National Health Service Survey data from 2003 to 2013 (age range: ≥15 years) showed a slow decrease in tobacco smoking from 48.4% to 47.2% among Chinese men and from 3.1% to 2.7% among Chinese women [3]. To address the paucity of data since 2013, using 5 consecutive nationally representative cross-sectional surveys, Zhang and colleagues report that the prevalence of tobacco smoking has decreased among men from 58.4% to 50.8% between 2007 and 2018, with the prevalence of tobacco smoking leveling off at about 2% for women during this period. Additionally, diverging trends were observed between urban and rural areas and across 31 provinces in China.

An important finding by Zhang and colleagues is that the prevalence of tobacco smoking has increased among rural men born after 1990, from 40.2% in 2007 to 52.1% in 2018. If effective smoking prevention or cessation measures are not successfully implemented in this specific population, the overall decline observed for men is in danger of slowing or even reversing.

Zhang and colleagues also observed significant geographic variation in the prevalence of tobacco smoking across 31 provinces in China in 2018. Smoking cessation services should be comprehensively strengthened in China in consideration of low cessation rate among current smokers (approximately 15%) and differences in service ability of smoking cessation clinics [4], which differ in their availability by region (urban versus rural), as well as by province.

Although the tobacco smoking prevalence among Chinese adults has decreased during the past 2 decades, e-cigarette use has increased in recent years. Data from 2 nationally representative cross-sectional surveys showed that the prevalence of past 30-day e-cigarette use among Chinese adults increased from 1.3% in 2015 to 1.6% in 2019, a relative increase of approximately 23% [5]. There were also diverse trends in urban and rural regions, with the prevalence changing from 1.6% to 2.1% in urban region and 1.0% to 1.1% in rural region. E-cigarettes are sometimes used as a substitute for smoking cessation and are erroneously considered by some to be less harmful to health than traditional burnt cigarettes. However, evidence has suggested adverse impacts of the toxic constituents of e-cigarettes on endothelial function and subsequent cardiovascular disease [6], and thus e-cigarettes are not an ideal alternative to aid in cessation of smoking tobacco cigarettes.

Zhang and colleagues acknowledge that they were unable to investigate smoking trends among children and adolescents because their surveys did not include the pediatric population. Data from China National Youth Tobacco Surveys have shown a decrease in the prevalence of current cigarette use among adolescents aged 13 to 15 years from 5.9% to 3.9% from 2014 to 2019 [7], whereas the prevalence of e-cigarette use has increased from 1.2% to 2.7% over the same period.

Thus, the trends in prevalence of tobacco smoking and e-cigarette use appear similar in the pediatric setting compared with adults in China. However, while similar to adults, the prevalence of e-cigarette use seems a bit higher. These abovementioned findings highlight the necessity to strengthen tobacco control efforts among young adolescents.

In 2005, the Chinese government ratified the World Health Organization Framework Convention for Tobacco Control (WHO FCTC). An aim of the study by Zhang and colleagues was to see whether the ratification of the WHO FCTC since 2005 had played a key role in trend of smoking prevalence among Chinese adults. Unfortunately, there is no substantial progress in implementation of the WHO FCTC in China. Although about 22 provincial capital cities and 4 municipalities in China have established local regulations on tobacco control, there is still no unified national legislation on tobacco control. Additionally, the executive abilities of 26 cities in China are largely different, except for the good execution effect (i.e., the ability to enact or enforce tobacco control policies) in some big cities such as Beijing and Shanghai. For example, Shanghai has made substantial progress on tobacco control. In March 2010, Shanghai implemented the Regulation on Smoking Control in Public Places in Shanghai Municipality policy, which limited smoking in public places [8]. The regulation was updated in March 2017, which achieved a complete indoor smoking ban. In 2018, the lowest prevalence of tobacco smoking in men across China was observed in Shanghai (34.8%) as reported by Zhang and colleagues.

To effectively control tobacco smoking in China, national or regional/provincial legislation and successful implementation of tobacco control policies are needed. Brazil is one example of successful implementation of tobacco control, mainly attributable to the strict national regulation on tobacco control and high adherence to the WHO FCTC [9]. Policies and regulations enacted in China should include an increase on tobacco taxation, making harm warnings on cigarette packs more prominent or in combination with plain packaging, programs aimed at reducing the rate of smoking initiation, an increase in smoking cessation services, complete and up-to-date prohibition of tobacco sponsorship and advertising (especially for supervision of online tobacco marketing), and smoke-free home and public places that are recommended by the WHO FCTC [10]. In all, the more recent update on a slowing decrease in smoking prevalence provides a more evidence in support for strong tobacco control policies to be put into place in China.
